# Functional roles of circular RNAs during epithelial-to-mesenchymal transition

**DOI:** 10.1186/s12943-019-1071-6

**Published:** 2019-09-16

**Authors:** Bing-Qing Shang, Min-Le Li, Hao-yu Quan, Ping-Fu Hou, Zhong-Wei Li, Su-Fang Chu, Jun-Nian Zheng, Jin Bai

**Affiliations:** 1 Cancer Institute, Xuzhou Medical University, 84 West Huaihai Road, Xuzhou, 221002 Jiangsu Province China; 2 Center of Clinical Oncology, Affiliated Hospital of Xuzhou Medical University, Xuzhou, 221002 Jiangsu Province China

**Keywords:** Epithelial-to-mesenchymal transition, CircRNAs, Metastasis, Biomarker

## Abstract

Cancer has become a major health issue worldwide, contributing to a high mortality rate. Tumor metastasis is attributed to the death of most patients. Epithelial-to-mesenchymal transition (EMT) plays a vital role in inducing metastasis. During EMT, epithelial cells lose their characteristics, such as cell-to-cell adhesion and cell polarity, and cells gain motility, migratory potential, and invasive properties to become mesenchymal stem cells. Circular RNAs (circRNAs) are closely associated with tumor metastasis and patient prognosis, as revealed by increasing lines of evidence. CircRNA is a type of single-stranded RNA that forms a covalently closed continuous loop. CircRNAs are insensitive to ribonucleases and are widespread in body fluids. This work is the first review on EMT-related circRNAs. In this review, we briefly discuss the characteristics and functions of circRNAs. The correlation of circRNAs with EMT has been reported, and we discuss the ways circRNAs can regulate EMT progression through EMT transcription factors, EMT-related signaling pathways, and other mechanisms. This work summarizes current studies on EMT-related circRNAs in various cancers and provides a theoretical basis for the use of EMT-related circRNAs in targeted management and therapy.

## Background

Epithelial-to-mesenchymal transition (EMT) is an important cellular event widely involved in physiologic and pathologic processes, including embryonic organ development [1, 2], wound healing, and tumor metastasis [3, 4]. During EMT, epithelial cells lose their characteristics, such as cell-to-cell adhesion and cell polarity. Instead, cells gain motility, migratory potential, and invasive properties, thereby becoming mesenchymal stem cells. The understanding of the mechanism of EMT progression has improved. The expression of epithelial markers, such as E-cadherin (E-cad), decreases and that of mesenchymal markers, such as N-cadherin (N-cad), vimentin, and claudin, increases during EMT progression. Multiple EMT transcription factors (EMT-TFs), including the Snail, Twist, and Zeb families, converge into EMT regulation. In addition to EMT-TFs, several signaling pathways have been identified, i.e., the transforming growth factor β (TGF-β)/Smad, Wnt/β-catenin, and Hedgehog signaling pathways. Noncoding RNAs can modulate EMT through EMT-TFs and EMT-related signaling pathways. Noncoding RNAs comprise a wide range of RNAs with different characteristics. This review mainly discusses the modulation of circRNAs in EMT progression.

**Table 1:** Online circRNA databases

Name	Description of the database	Website address	Reference
circBase	Database of circRNAs from different species	http://www.circbase.org/	[31]
circNet	Database of circRNAs originated from transcriptome sequencing data	http://circnet.mbc.nctu.edu.tw/	[32]
starBase	Database of circRNA-miRNA interactions	http://starbase.sysu.edu.cn/	[33]
deepBase	Database of lncRNAs and circRNAs	http://rna.sysu.edu.cn/deepBase/	[34]
circ2Traits	Database of circRNA-related diseases	http://gyanxet-beta.com/circdb/	[35]
TSCD	Database of tissue-specific circRNA	http://gb.whu.edu.cn/TSCD/	[36]
CSCD	Database of cancer-specific circRNA	http://gb.whu.edu.cn/CSCD	[37]

**Table 2:** EMT-related circRNAs acting through WNT signaling

circRNA	Expression pattern	Interaction with WNT signaling	Sponged miRNA	Cancer type	Reference
CircRNA-100290	Up	Activate	miR-516b	CRC	[74]
CircRNA-NEK6	Up	Activate	miR-370-3p	TC	[75]
Circ_0000177	Up	Activate	miR-638	Glioma	[76]
Circ_CBFB	Up	Activate	miR-607	CLL	[77]
Circ_0001946	Up	Activate	miR-13a-5p	LAC	[94]
CircRNA_102171	Up	Activate	–	PTC	[99]
Circ_0006427	Down	Inhibit	miR-6783-3p	LUAD	[80]
Circ_0000523	Down	Inhibit	miR-31	CRC	[81]
Circ-ITCH	Down	Inhibit	miR-214	Glioma	[90]
Circ-ITCH	Down	Inhibit	miR-214, miR-17	TNBC	[89]
Circ_0002052	Down	Inhibit	miR-1205	OS	[85]

*CRC* Colorectal cancer, *TC* Thyroid cancer, *CLL* Chronic lymphocytic leukemia, *LAC/LUAD* Lung adenocarcinoma, *PTC* Papillary thyroid cancer, *TNBC* Triple-negative breast cancer, *OS* Osteosarcoma

**Fig. 1:**
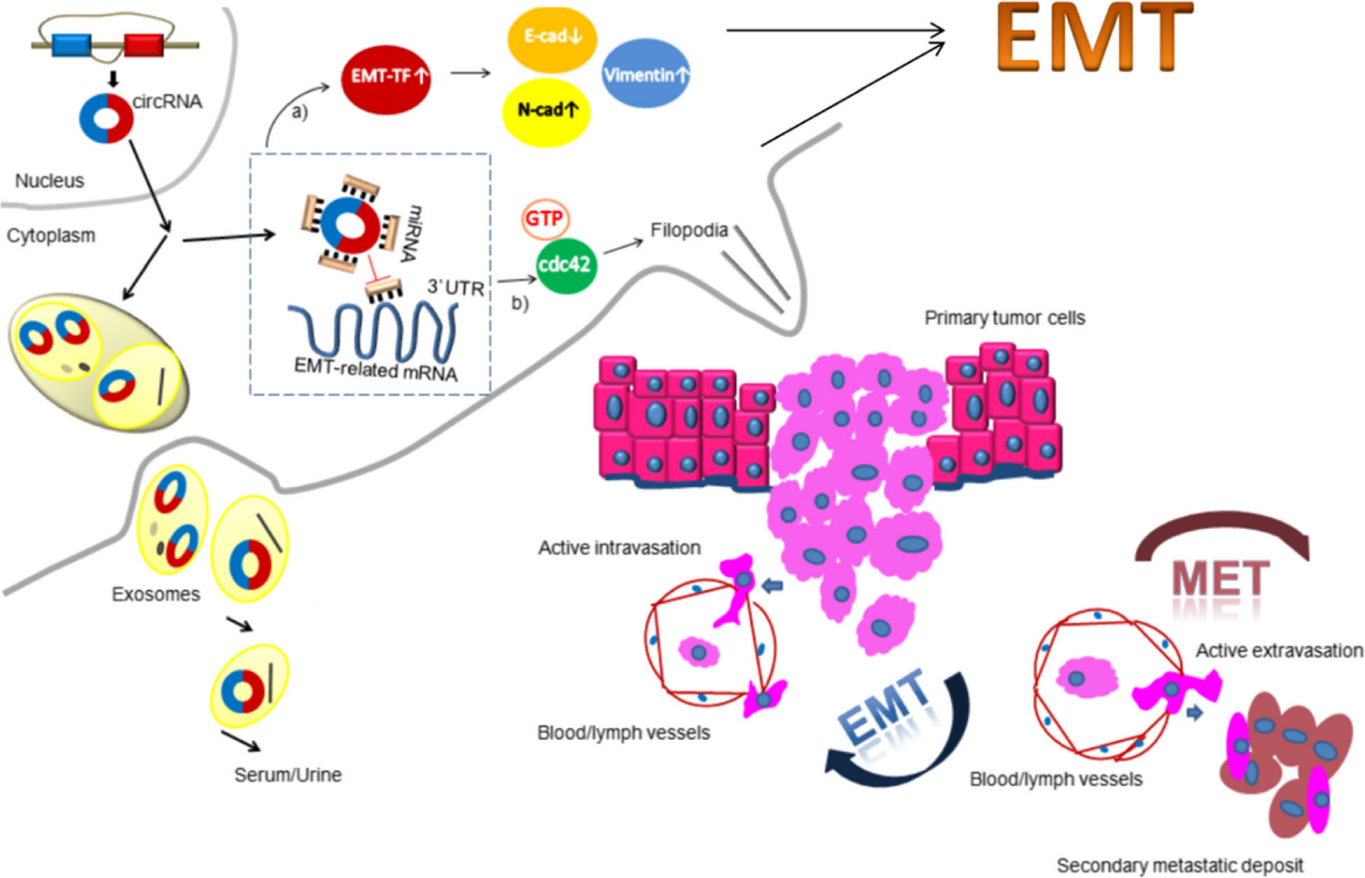
CircRNA plays an important role in tumor metastasis. During EMT, circRNA absorbing miRNA attenuates the inhibition of the miRNA on EMT-related genes, such as EMT-TF/E-cad, N-cad, vimentin and cdc42. As a result, epithelial cells lose their characteristics, such as cell-to-cell adhesion and cell polarity. Instead, the cells gain motility, migratory potential, and invasive properties, becoming mesenchymal stem cells. In addition, EMT triggers primary tumor cells to enter the circulatory or lymphatic system. Then, circulating tumor cells are transported to distant tissues or organs to form secondary metastatic deposits through EMT. Of note, circRNAs secreted as exosomes are detected in serum/urine, which provides a novel strategy for the diagnosis and treatment of cancer

Circular RNA (circRNA) is a type of single-stranded RNA that forms a covalently closed continuous loop. In circular RNAs, unlike the known linear RNAs, the 3′ and 5′ ends normally present in an RNA molecule are linked. As a result, circRNAs are insensitive to ribonucleases. In 1976, the first circRNA was found in an RNA virus [5, 6]. In 1991, Nigor et al. [7] observed circRNAs in eukaryotes. CircRNAs were considered as an accidental byproduct until Jeck WR found two types of circRNA formations in 2013 [8]. With the rapid development of sequencing technology, the characteristics and functions of circRNAs have been revealed. Numerous circRNAs have been identified in humans. circRNAs exist widely in tissues, serum, and urine. CircRNAs have been identified as a determinant factor in many human diseases, including cardiovascular system diseases, neurological disorders, and cancer. Moreover, several studies have demonstrated that circRNAs can serve as prognostic biomarkers because of their characteristics [9–13].

This review summarizes current studies on EMT-related circRNAs in various cancers and provides a theoretical basis for the use of EMT-related circRNAs in targeted management and therapy.

## Biogenesis of circRNAs

CircRNAs are classified into three categories: exon circRNA (ecRNA), circular intron RNA (ciRNA), and exon–intron circRNA (EIciRNA). In 2013, Jeck proposed that exon skipping and intron pairing are events that narrow the distance between splice sites and facilitate the back-splicing of pre-mRNA. This phenomenon results in circRNAs that lack 3′ and 5′ ends. In ecRNA and EIciRNA formation, circRNAs are linked by a 3′–5′ phosphodiester bond, whereas ciRNAs are linked by a 2′–5′ phosphodiester bond [14]. In general, the generation of linear RNA involves removing introns and linking exons in sequence. The competitive relationship between linear splicing and back-splicing determines the fate of hnRNA. As such, circRNA formation is precisely controlled. The two introns flanking the circularized exons, which have been found to be enriched in Alu repeats, can increase the efficiency of circularization [8, 15]. Some proteins regulate the biogenesis of circRNA, including RNA binding proteins (RBPs) and the spliceosome [16, 17]. The regulation of circRNA formation by proteins is complex and consists of positive and negative modulations. For example, quaking can promote circRNA biogenesis by binding to specific sites in the intron region [18]. In contrast, some proteins are negatively associated with the formation of circRNAs. Adenosine deaminase acting on RNA-1 (ADAR1), as an RNA-editing factor, reduces circRNA expression. Several studies have found that ADAR1 negatively modulates circRNA formation by interacting with DExH-box helicase 9 (DHX9) [19] and androgen receptor [20].

## Function of circRNAs

After their discovery, circRNAs were misunderstood as the result of splicing errors. With the development of gene sequencing and other technologies, studies have found that circRNAs are involved in biological processes by being translated into peptides [21–24], binding to RBPs [25, 26], and sponging miRNAs [27, 28]. Among these processes, circRNAs most commonly act as miRNA sponges in tumor cells. CircRNAs have many miRNA binding sites. CircRNAs are often negatively correlated with miRNAs and repress miRNA activity. For example, circPRMT5 facilitates urothelial carcinoma of the bladder (UCB) cell metastasis by sponging tumor-suppressor miR-30c [9]. However, Piwecka M et al. illustrated that circRNAs can positively modulate miRNAs and decrease miRNA downstream genes [29]. CircRNAs can interact with RBPs, thus affecting nuclear translocation and modulating the targeted pathway. CircRNAs can also play a protein-coding role. Several studies on protein-coding circRNAs, which can be translated into peptides by binding to ribosomes, have been performed [21–24].

## Databases of circRNAs

With the development of high-throughput sequencing technology, numerous circRNAs have been found and reported in databases [30] (Table 1). The circBase database mostly contains circRNAs of different species, such as human, mouse, and *Latimeria* organisms*.* Sequence information and evidence supporting the expression of circRNAs can be searched and downloaded [31]. The CircNet database includes transcriptome sequencing datasets and data on expression in specialized tissues [32]. The CircNet and starBase databases also provide data on networks between circRNAs, miRNAs, and targeted genes [32, 33]. Information on circRNAs and lncRNAs can also be found in the deepBase database. This database contains the sequencing datasets of a large amount of circRNAs [34]. The Circ2Traits database includes information on RNA-related diseases and RNA traits. It has classified thousands of circRNAs connected to hundreds of diseases [35]. Tissue-specific circRNAs are mostly found in the tissue-specific circRNA database (TSCD). TSCD contains data on the human and mouse genomes [36]. The cancer-specific circRNA database is the first database that fully comprises cancer-specific circRNAs. This database may provide a meaningful strategy for the control of tumor cells [37].

## Contribution of circRNAs to epithelial-to-mesenchymal transition regulation

Cell–cell contact and cytoskeletal proteins undergo changes during EMT. A decrease in the expression of the epithelial marker E-cad is possibly a significant event, whereas the levels of mesenchymal markers, such as N-cad and vimentin, increase [38–41]. Several EMT-TFs, such as the Snail, Twist, slug, and ZEB families, are known regulators during EMT. They can bind to E-boxes to regulate EMT markers [42–46]. Some signaling pathways, particularly the Wnt/β-catenin, TGF-β/SMAD, and Notch pathways, may induce EMT [39, 47–49]. Here, we provide up to date information on circRNAs involved in EMT mechanisms.

### CircRNA control of EMT-TFs

#### Regulation of snail by circRNAs

Several circRNAs are potentially involved in regulating EMT-TFs, which are considered fundamental players in EMT. Snail is an EMT-TF and is positively correlated with EMT progression [50, 51]. For example, circRNA_0084043 is significantly upregulated in melanoma tissues. Bioinformatics analysis and luciferase reporter assays have confirmed that circRNA_0084043 directly interacts with miR-153-3p and that Snail is directly targeted by miR-153-3p. Therefore, circRNA_0084043 overexpression upregulates Snail expression and promotes EMT progression. The circRNA_0084043/miR-153-3p/Snail axis is the first discovery in melanoma related to circRNA. CircRNAs provide a potential new use for targeted therapy for melanoma [52]. A human circRNA microarray has been used to explore the upregulated circRNA_000284 in cervical cancer cells. Similarly, miR-506 has been identified as a circRNA-000284-associated miRNA by performing a luciferase reporter assay and through anti-AGO2 RNA precipitation. In addition, Snail-2 has been confirmed as a direct target of miR-506. As a result, circRNA-000284 can indirectly increase Snail-2 expression and promote invasion, migration, and metastasis [53]. CircPRMT5 serves as a sponge for miR-30c in UCB, relieving the miR-30c-mediated repression of Snail-1. Increased circPRMT5 facilitates UCB cell metastasis by downregulating Snail-1-related E-cad and increasing vimentin and N-cad [9]. Studies on circRNAs and Snail are few, and additional studies on the mechanism of the regulation of Snail by circRNAs are still needed.

#### Regulation of twist on vimentin by circRNAs

Twist as an important EMT-TF that directly or indirectly governs the transcription of EMT-associated genes, thereby decreasing epithelial markers, such as E-cad, and increasing mesenchymal cell markers, including vimentin [54]. Cullin2 (Cul2) is a core component of multiple ElonginB/C-CUL2/5-SOCS-box protein (ECS) E3 ubiquitin–protein ligase complexes that mediate the ubiquitination of target proteins [55, 56]. A study has validated that Twist1 promotes Cul2 transcription by binding to the Cul2 promoter and increases the pre-mRNA levels of Cul2. As a result, Twist1 upregulates Cul2 circRNA (circRNA_10720) expression. CircRNA_10720 is positively associated with EMT and promotes metastasis in hepatocellular carcinoma (HCC) cells. CircRNA_10720 regulates vimentin by sponging a series of miRNAs. This research hypothesizes that miR-490-5p is the main absorbed miRNA that regulates vimentin, as shown by a luciferase reporter assay. CircRNA_10720 knockdown counteracts the effect of Twist1 by promoting metastasis through the upregulation of vimentin [57]. This process provides a novel mechanism of how Twist regulates vimentin via a circRNA.

### TGF-β/Smad pathway-related circRNAs

Previous studies have demonstrated that TGF-β can enhance the EMT process. The TGF-β/Smad pathway has been demonstrated to be a positive regulator in the growth and metastasis of various cancer types [58, 59]. Wang L et al. reported that circPTK2 inhibits non-small-cell lung carcinoma (NSCLC) metastasis by disrupting oncogenic miR-429 and miR-200b-3p and promoting transcriptional intermediary factor 1γ (TIF1γ) expression [60]. TIF1γ, which is considered as a negative regulator of the TGF-β/Smad pathway [61, 62], represses the TGF-β-related EMT. Zeng K et al. demonstrated that the high expression level of circANKS1B can be indicative of a poor prognosis in triple-negative breast cancer (TNBC). The upregulated circANKS1B has been implicated in promoting breast cancer invasion and metastasis. The pro-metastatic effect of circANKS1B is mediated through the interaction with miR-148a-3p and miR-152-3p, thereby increasing upstream transcription factor 1 (USF1) expression [63]. USF1 binds to TGF-β1 in murine tissues [64]. Zeng K et al. confirmed that upregulated USF1 can interact with TGF-β1 in TNBC tissues to substantially upregulate the expression of vimentin, p-Smad2, and p-Smad3 but significantly decrease the expression of E-cad. Moreover, circANKS1B expression is regulated by USF1. USF1 overexpression upregulates circANKS1B expression via the splicing factor Epithelial splicing regulatory protein 1 (ESRP1), showing that a positive feedback regulatory loop is formed between circANKS1B, USF1, and ESRP1 [63]. Splicing factors (SFs) can also regulate circRNA formation and EMT progression by inserting SF-binding motifs into flanking introns, which has been demonstrated in several research reports [18, 63, 65, 66].

### Wnt pathway-related circRNAs

In general, the Wnt pathway can be classified as a canonical pathway (Wnt/β-catenin pathway) and a noncanonical pathway (Wnt/PCP and Wnt/Ca pathways). Here, we mainly discuss the canonical Wnt/β-catenin pathway. In brief, in the absence of Wnt signaling, β-catenin, Axin, glycogen synthase kinase-3, adenomatous polyposis coli (APC), and casein kinase 1 form a destruction complex in the cytoplasm in which the initially phosphorylated and subsequently ubiquitinated β-catenin is degraded by the proteasome. The presence of Wnt ligands blocks the formation of this complex. As a result, β-catenin accumulates to a certain level in the cytoplasm and then translocated into the nucleus to activate the Wnt-targeted genes [67–70]. The interaction between circRNAs and the Wnt/β-catenin pathway to promote cancer progression has been demonstrated (Table 2).

Frizzled gene family proteins (FZDs) are important cell surface receptors in the Wnt/β-catenin signaling pathway and consists of FZD1/2/3/6/7, FZD5/8, FZD4, and FZD9/10 subfamilies. FZDs are positive membrane receptors in the Wnt/β-catenin pathway and are significantly associated with cancer progression [71–73]. Guan F et al. demonstrated the obvious overexpression of circRNA_100,290 and FZD4 in colorectal cancer (CRC) and found their target relationship with miR-516b. CircRNA_100,290 not only increases proliferation and metastasis by regulating FZD4 but also upregulates the expression of MYC, CCD1, CCD2, TCF7, and SOX4, which are target genes of the Wnt/β-catenin signaling pathway [74]. In thyroid cancer, circNEK6 is a competing endogenous RNA of FZD8 that absorbs miR-370-3p, resulting in the activation of the Wnt/β-catenin signaling pathway. The upregulation of circNEK6 enhances the expression of c-myc and CCD1, whereas silencing FZD8 abrogates their expression [75]. Chen et al. demonstrated that hsa_circ_0000177 is a miR-638 sponge that targets FZD7. Furthermore, hsa_circ_0000177 activates the Wnt/β-catenin pathway and promotes the growth of glioma cells by enhancing FZD7 expression [76]. Xia et al. showed that circ-CBFB ultimately leads to CLL progression via the FZD3-induced activation of Wnt/β-catenin signaling by sponging miR-607 [77].

Dickkopf-1 (DKK1) can specifically bind to LRP5/6, thereby interfering with the formation of the Wnt-LRP5/6-FZD complex and inhibiting the downstream pathway [78, 79]. Yao et al. illustrated that the significantly downregulated circ_0006427 is involved in the tumorigenesis of lung adenocarcinoma (LUAD) by releasing miR-6783-3p and by activating the Wnt signaling pathway through DKK1 knockdown. Circ_0006427 downregulation or DKK1 knockdown promotes cell migration and invasion [80]. Y. Jin et al. demonstrated a significant decrease in hsa_circ_0000523 and DKK1 in CRC and identified their target correlation with miR-31. A decreased DKK1 level not only enhances β-catenin in the nucleus but also promotes CRC proliferation [81].

APC is considered a negative regulator in the Wnt/β-catenin signaling pathway and is associated with the β-catenin destruction complex [82–84]. Hsa_circ_0002052 and APC2 are obviously decreased in osteosarcoma (OS) and are strongly correlated. As a result, the downregulation of hsa_circ_0002052 promotes proliferation and metastasis through the Wnt/β-catenin pathway. MiR-1205 has been identified as a common target miRNA of hsa_circ_0002052 and APC2 and is upregulated in OS. Hsa_circ_0002052 also provides a novel therapeutic target for OS therapy [85].

ITCH is a E3 ubiquitin ligase enzyme and can inactivate the Wnt/β-catenin pathway by degrading phosphorylated dishevelled Dvl [86–88]. Dvl can bind to Axin, which is an important component of the β-catenin destruction complex, to positively modulate the β-catenin level. Several studies have demonstrated the positive relationship between circ-ITCH and ITCH. In TNBC, circ-ITCH exhibits breast tumor inhibition and can promote the expression of ITCH tumor suppressor by absorbing miR-214 and miR-17 [89]. Feng Li et al. illustrated the anti-oncogenic effect of circ-ITCH on glioma. Decreased circ-ITCH and ITCH can promote migration and invasion by sponging miR-214. In this mechanism, miR-214 and miR-17 can bind to the 3′UTR of ITCH to enhance ITCH expression [89, 90].

Sirtuin 1 (SIRT1) exerts diverse effects on tumor cells as an oncoprotein or tumor suppressor [91–93]. Yao Y et al. demonstrated that circ_0001946 expression is increased in lung adenocarcinoma. Circ_0001946 upregulates SIRT1 and activates the Wnt/β-catenin pathway in LAC by targeting miR-135a-5p, thus promoting LAC progression [94]. However, the mechanism involving circRNA and SIRT1 remains largely unknown.

Previous studies have found that catenin beta-1 (CTNNBIP1) is a negative regulator in the Wnt/β-catenin pathway and can interact with β-catenin. Mechanistically, increasing evidence has shown that the CTNNBIP1/β-catenin interaction can prevent the formation of the β-catenin/TCF/LEF complex. As a result, it prevents the activation of the Wnt/β-catenin signaling pathway [95–98]. The upregulated circRNA_102171 facilitates the malignant behavior of papillary thyroid cancer by regulating the CTNNBIP1-mediated activation of the Wnt/β-catenin pathway. CircRNA_102171 overexpression enhances the β-catenin/TCF/LEF interaction while blocking the association between CTNNBIP1 and β-catenin. Nevertheless, how circRNA_102171 modulates CTNNBIP1 still needs further studies [99].

### Regulation of cell adhesion molecules and cytoskeletal proteins by circRNAs

In addition to the regulation exerted by circRNAs at the level of transcription factors and EMT-related signaling pathways, certain circRNAs function by directly or indirectly regulating the expression of cell adhesion molecules and cytoskeletal proteins. Such deregulated circRNAs can act either as tumor suppressors or oncogenes to control cell proliferation, migration and metastasis. For example, circ_0058063 promotes bladder cancer progression by sponging miR-145-5p and regulating CDK6 expression [100]. In HCC, the circRNA circMTO1 (mitochondrial translation optimization 1 homologue) significantly downregulated and circMTO1 overexpression suppressed HCC cell proliferation and invasion by sponging oncogenic miR-9. Moreover, circMTO1 is an important circRNA in bladder cancer tissue. A decreased circMTO1 level was positively correlated with bladder cancer cell metastasis. Further research found that circMTO1 was able to sponge miR-221, and ectopic expression of circMTO1 negatively regulated the E-cad/ N-cad pathway to inhibit bladder cancer cell EMT by competing for miR-221 [101].

Several circRNAs have been demonstrated to regulate vimentin transcription. For instance, Tao Wang et al. reported on the role of circP4HB in NSCLC. In NSCLC, circP4HB levels are significantly higher than in healthy lung. Of note, vimentin has a documented role in promoting EMT and metastasis in NSCLC as a regulator of cell to cell adhesion and cell motility. Interestingly, circP4HB can positively regulate vimentin and enhance EMT and metastatic disease through miR-133a-5p sequestration [102]. A recent circRNA profiling study showed that circRBM23 expression was upregulated, whereas miR-138 expression was decreased, in HCC tissues. Downregulation of circRBM23 decreased cell viability, proliferation, and migration and promoted the expression of miR-138 and its related target genes vimentin and CCND3 [103]. Thus, the circRNA-miRNA-vimentin regulatory axis in EMT is important for tumor invasion and metastasis.

Protocadherins (PCDHs) are classified as the largest subgroup within the cadherin superfamily of calcium-dependent cell-cell adhesion molecules that are mainly expressed in the nervous system and have been implicated in neural cell-cell interactions. Little is known about the functions of PCDHs, but some members, such as PCDH8 and PCDH10, have been shown to suppress tumor activity [104–107], suggesting that PCDHs may act as tumor suppressors by influencing tumor growth and metastasis. In prostate cancer (PCa), Zhan Yang et al. have shown that an Amotl1-derived circRNA termed circAMOTL1L, is downregulated in PCa and that low expression of circAMOTL1L facilitates PCa cell migration and invasion by downregulating E-cad and upregulating vimentin, which leads to EMT and PCa progression. Mechanistically, the study demonstrated that circAMOTL1L serves as a sponge for binding miR-193a-5p in PCa cells, relieving the miR-193a-5p-mediated repression of the Pcdha gene cluster, which is a subset of PCDHs [108].

### Regulation of cell motility and metastasis by circRNAs through rho GTPases

Carcinoma cells that undergo EMT reorganize the epithelial actin cytoskeleton into one with actin stress fibers and form migratory organelles, such as lamellipodia and filopodia. In this way, cells acquire directional motility and metastatic potential [109]. Rho GTPases belong to the group of small G proteins that cycle between an inactive GDP-bound state and active GTP-bound state. Among the members of the Rho GTPase family, RhoA, cdc42 and Rac have been studied in most detail. RhoA facilitates the formation of actin stress fibers, while cdc42 and Rac1 generally assist in filopodia and lamellipodia formation. Rho-kinase (ROCK), as the downstream effector of RhoA, induces myosin light chain (MLC) phosphorylation by inhibiting MLC phosphatase to enhance myosin contractility. ROCK also activates LIM kinase (LIMK) and then represses cofilin. The Rho/ROCK/MLC/myosin and Rho/ROCK/LIMK/cofilin pathways all facilitate stress fiber formation. Moreover, actin polymerization dependent on mDia is an essential factor for the assembly of stress fibers [110, 111]. CircHIAT1 expression was suppressed by androgen receptor (AR) in clear cell renal cell carcinoma (ccRCC). Interestingly, circHIAT1 could stabilize the expression of miR-195-5p/29a-3p/29c-3p as a reservoir. It has been demonstrated that the downregulating miR-195-5p/29a-3p/29c-3p targets the cdc42 3′-UTR to enhance the migration and invasion of ccRCC cells. Notably, upregulation of AR-targeting cdc42 significantly promoted filopodia formation, while the increase could be partially reversed by CircHIAT1 overexpression [112]. Jie Li et al. investigated the role of circ-IARS in pancreatic cancer tissues. Circ-IARS was secreted by pancreatic cancer cells and upregulated in plasma exosomes. Then, circ-IARS enters HUVECs through exosomes. In this study, upregulation of circ-IARS in HUVECs changed their biological functions. It has been demonstrated that circ-IARS sponges miR-122 to increase RhoA activity and F-actin expression and reduce ZO-1 expression. As a result, circ-IARS/RhoA modulates the permeability of the endothelial monolayer to promote metastasis [113].

### Other pathways related to circRNAs

High-mobility group box 1 (HMGB1) is considered a chromatin-binding factor that regulates HCC progression [114–116]. HMGB1 knockdown can significantly attenuate the migration and invasion behavior [117, 118]. Li S et al. demonstrated the correlation between a circRNA and the HMGB1/RAGE pathway. Upregulating circRNA_101,368 suppresses miR-200a expression, thus enhancing the metastasis of HCC cells by activating the miR-200a-mediated HMGB1/RAGE pathway [119].

MiR-7 is considered a tumor cell suppressor whose dysregulation facilitates malignant behavior [120, 121]. The experimental basis for the targeted treatment from the perspective of the circRNA CDR1as/miR-7 pathway has been validated in several cancer cells. Su C et al. demonstrated that the remarkably increase in ciRS-7 (circRNA CDR1as) induces the metastasis of NSCLC cells through the miR-7 downstream NF-κB pathway [122]. Furthermore, the circRNA CDR1as/miR-7 pathway regulates the EMT phenotype. Furthermore, a miR-7 inhibitor can enhance the metastatic ability of HCC and OS cells [123, 124].

Yu J et al. linked the circRNA cSMARCA5/miR-17-3p/miR-181b-5p/TIMP3 regulatory pathway to EMT in HCC [125]. TIMP3 has been reported to be a tumor cell suppressor in several studies and inactivates the EMT program [126, 127]. It has been found that the decrease in circRNA cSMARCA5 facilitates HCC cell migration and metastasis and downregulates TIMP3 transcription by absorbing miR-17-3p and miR-181b-5p [125].

Human mesenchymal stem cells (MSCs) are multipotent cells that possess the ability to self-renew and differentiate into mesodermal lineage cells [128, 129]. Due to this differentiation potential and other properties to regenerate injured tissues indirectly via growth factor secretion and immunomodulation, MSCs hold promise for regenerative medicine. A full understanding of the molecular mechanisms that regulate the maintenance of human MSC identity and their uncommitted state is helpful for improving therapeutic efficacy. Stem cell plasticity and identity are controlled by master regulatory genes and complex circuits involving noncoding RNAs [130–132]. Alessandro Cherubini et al. showed that compared to that in differentiated mesodermal cells, circFOXP1 levels were enriched in MSCs. The authors demonstrated a direct interaction between circFOXP1 and miR-17-3p/miR-127-5p, which resulted in the modulation of the EGFR and noncanonical Wnt pathways. They found a regulatory role for circFOXP1 as a gatekeeper of pivotal stem cell molecular networks [133]. Yanjie Wang et al. demonstrated that circRNA_014511 could affect the expression of P53, regulate cell apoptosis and cell cycle arrest, and influence the radiosensitivity of bone marrow mesenchymal stem cells (BMMCs) by adsorbing miR-29b-2-5p. Furthermore, Yan-Jing Zhu et al. found that circZKSCAN1 suppressed cell stemness in HCC by regulating the function of the RBP fragile X mental retardation protein (FMRP), whose downstream target gene is cell cycle and apoptosis regulator 1 (CCAR1) and showed that CCAR1 acts as a coactivator of the Wnt/β-catenin signaling pathway and upregulates cell stemness [134].

## Conclusions and future perspectives

Metastasis is an important factor causing malignant tumor behavior. A number of circRNAs have been correlated with tumor cell EMT regulation (Fig. 1). CircRNAs are closely associated with tumor progression and patient prognosis. However, unlike studies on other noncoding RNAs, further efforts are needed to produce several EMT-related circRNAs. Furthermore, this work is the first review on the associations between circRNAs and EMT. The exact mechanisms of how circRNAs regulate EMT remain unclear. CircRNA-based diagnostics and treatments have many challenges. The former involves a low circRNA expression level and advanced detection measures. The latter includes the requirement of safety and effectiveness and the absence of off-target effects. CircRNAs are promising novel cancer markers because of their ribonuclease insensitivity and widespread expression in body fluids. Some studies have suggested that exosomal circRNAs are stable. Thus, future studies should further develop several EMT-related circRNAs associated with various types of cancers and characterize the function of circulating circRNAs, especially exosomal circRNAs, to make a potent therapy to directly target circRNAs. Overall, identifying the specific functions and mechanisms of circRNAs in EMT will provide novel and powerful perspectives on tumor management.

## Data Availability

Not applicable.

## Abbreviations

ADAR1: Adenosine deaminase acting on RNA-1
APC: Adenomatous polyposis coli
AR: Androgen receptor
BMMCs: Bone marrow mesenchymal stem cells
CCAR1: Cell cycle and apoptosis regulator 1
ccRCC: Clear cell renal cell carcinoma
circRNA: Circular RNA
ciRNA: Circular intron RNA
CRC: Colorectal cancer
CTNNBIP1: Catenin beta-1
Cul2: Cullin2
DHX9: DExH-box helicase 9
DKK1: Dickkopf-1
E-cad: E-cadherin
ecRNA: Exon circRNA
ECS: ElonginB/C-CUL2/5-SOCS-box protein
EIciRNA: Exon–intron circRNA
EMT: Epithelial-to-mesenchymal transition
EMT-TFs: EMT transcription factors
ESRP1: Epithelial splicing regulatory protein 1
FMRP: Fragile X mental retardation protein
FZDs: Frizzled gene family proteins
HCC: Hepatocellular carcinoma
HMGB1: High-mobility group box 1
LIMK: LIM kinase
LUAD: Lung adenocarcinoma
MLC: Myosin light chain
MSCs: Human mesenchymal stem cells
N-cad: N-cadherin
NSCLC: Non-small-cell lung carcinoma
OS: Osteosarcoma
Pca: Prostate cance
Pcdha: Protocadherin-α
RBP: RNA binding protein
ROCK: Rho-kinase
SFs: Splicing factors
SIRT1: Sirtuin 1
TGF-β: Transforming growth factor β
TIF1γ: The transcriptional intermediary factor 1γ
TNBC: Triple-negative breast cancer
TSCD: Tissue-specific circRNA database
UCB: The urothelial carcinoma of the bladder
USF1: The upstream transcription factor 1
